# Impaired effort allocation in schizophrenia

**DOI:** 10.1016/j.scog.2025.100378

**Published:** 2025-07-15

**Authors:** Elodie Blouzard, Fabien Cignetti, Florent Meyniel, Arnaud Pouchon, Mircea Polosan, Julien Bastin, Clément Dondé

**Affiliations:** a Univ. Grenoble Alpes, Inserm, U1216, CHU Grenoble Alpes, Grenoble Institut Neurosciences, 38000 Grenoble, France; b INSERM-CEA Cognitive Neuroimaging Unit (UNICOG), NeuroSpin Center, CEA Paris-Saclay, Gif-sur-Yvette, France Université de Paris, Paris 91191, France; c Institut de Neuromodulation, GHU Paris, Psychiatrie et Neurosciences, Centre Hospitalier Sainte-Anne, Pôle Hospitalo-Universitaire 15, Université Paris Cité, Paris 75015, France; d Adult Psychiatry Department, CHU Grenoble Alpes, 38000 Grenoble, France; e France Adult Psychiatry Department, Centre Hospitalier Alpes-Isère, F-38000 Saint-Egrève, France

**Keywords:** Amotivation, Negative symptoms, Schizophrenia, Dynamic effort allocation, Decision-making

## Abstract

**Background and hypothesis:**

Effort allocation is a crucial component of amotivation in schizophrenia. This study investigates the hypothesis that schizophrenia is associated with impairments in dynamic cost/benefit decision-making processes.

**Study design:**

We employed a modified version of the effort allocation task developed by Meyniel et al. (2013). Participants were asked to allocate effort during 30-s intervals to maximize their gains. We examined the effects of task difficulty and incentive levels on participants' effort allocation on a trial-by-trial basis.

**Study results:**

Individuals with schizophrenia (*N* = 25) showed decreased capacity to adapt dynamically to task parameters, as compared to healthy controls (N = 25). (1) Both populations increased the duration of each effort based on difficulty. Only healthy controls decreased rest duration based on incentive. The magnitude of these adaptations was significantly decreased in people with schizophrenia (difficulty: d = 1.25, incentive: d = 0.91). (2) Both groups decreased effort re-initiations with increasing difficulty with significant differences in the magnitude of adaptation between groups. (3) Participants with schizophrenia spent less time exerting effort above the required threshold, resulting in lower overall gains compared to healthy controls (η^2^ = 0.17).

**Conclusions:**

Individuals with schizophrenia exhibit a selective impairment in effort-cost decision-making. This deficit may contribute to maladaptive behavior patterns characterized by suboptimal effort allocation and reduced goal-direct activities.

## 1 Introduction

Motivational impairments are core symptoms of schizophrenia and translate into significant losses in the daily functioning of patients (Jauhar et al., 2022). Re-initiating and sustaining effortful behaviors are essential for successful outcomes and daily goal-directed actions, such as social engagement, work tasks, or medication adherence. Hence, effort-cost decision-making is a dynamic process (Frömer and Shenhav, 2022; Rangel et al., 2008), yet previous studies on schizophrenia have primarily focused on static, pre-established, and often un-calibrated effort-related tasks (Blouzard et al., 2023).

In static effort-cost paradigms, abnormal effort-cost computations, characterized by overestimating the cost of effort, are hypothesized to contribute to motivational impairments and reduced goal-directed activities (Gold et al., 2015). Research has consistently shown that individuals with schizophrenia exhibit reduced willingness to expend effort for rewards, particularly for high rewards across tasks (reviewed in Blouzard et al. (2023)). This suggests an abnormal trade-off between the costs and benefits during decision making in schizophrenia. While most effort-based decision-making paradigms used in schizophrenia capture effort as a discrete choice (i.e., exert effort or not for a given reward), they fail to capture how effort is sustained and modulated over time, which is essential for understanding real-life motivation. In naturalistic settings, individuals must continuously regulate their effort output, balancing exertion and recovery across extended periods. The ability to repeatedly initiate effortful actions over time is essential for successful functioning in daily life—for instance, sustaining conversations during social events, performing repetitive tasks in assembly-line work, or adhering to regular medication schedules. Here, by examining effort and rest durations within a dynamic, self-paced paradigm, we aim to capture temporal aspects of cost computation, such as ability to adapt dynamically to task parameters, which are not accessible through static, binary-choice paradigms. This dynamic allocation of effort may reveal distinct motivational impairments in schizophrenia that are not captured by traditional effort-based decision-making tasks, which could contribute to functional difficulties in daily life. To our knowledge, how individuals with schizophrenia dynamically decide to either rest or work is unknown, although several factors suggest that their dynamic effort allocation may be impaired. Ecological momentary assessment studies have revealed that patients engage in fewer effortful activities and set lower effortful goals (Gard et al., 2014). Furthermore, pharmacological blockade of dopamine receptors, a mechanism implicated in motivational impairments in schizophrenia (Abi-Dargham, 2004; Okubo et al., 1997; Potkin et al., 2002), has been shown to reduce dynamic effort discounting over time in healthy individuals (Michely et al., 2020).

**Fig. 1:**
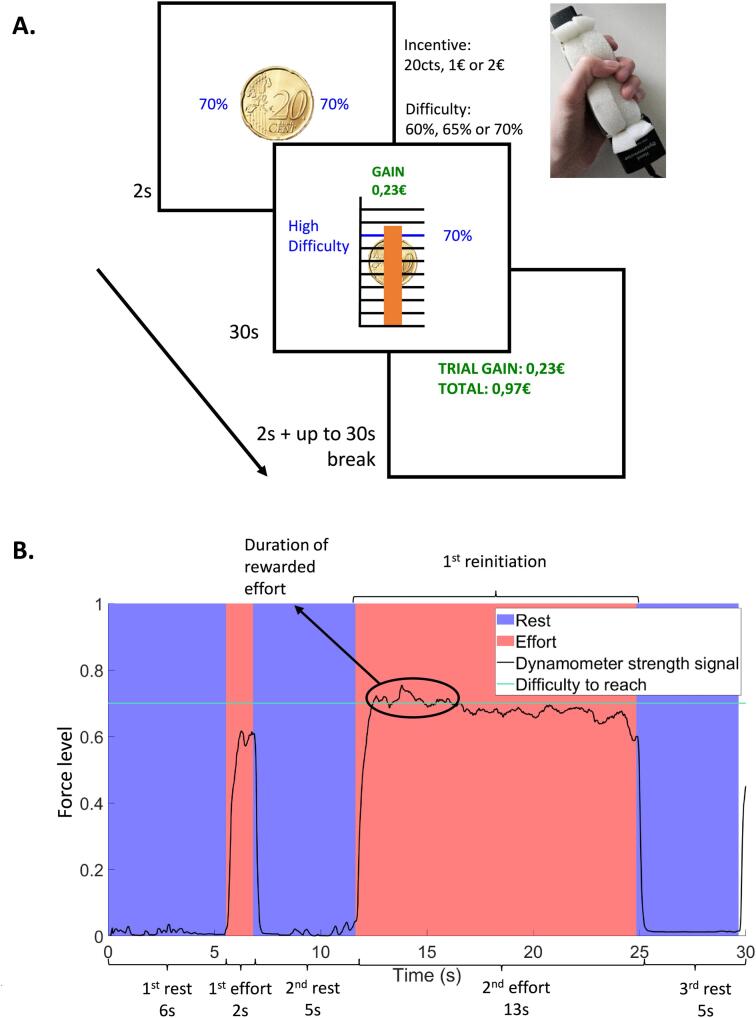
**Description of the dynamic effort allocation task.** **A. Experimental Paradigm:** Each trial began with a 2-s instruction screen displaying the incentive (a coin) and the difficulty level (as a percentage of maximal force). Participants had 30 s to accumulate as much money as possible by exerting effort using a manual dynamometer. A visual scale represented force levels, with the current trial's difficulty indicated on both sides and as a blue horizontal bar (70 % in the example). Gains were displayed and updated in real time (green text). When the subject exerted effort above the difficulty level, gains accumulated proportionally to the incentive level. The orange gauge represented the subject's real-time force. Between trials, participants had a break of 0 to 30 s. **B. Data Analysis:** The dynamometer force signal was extracted and epoched into effort/rest periods. The start and end of efforts were determined by sudden increases and decreases in the signal (see methods). The number of re-initiations was calculated by subtracting one from the total number of efforts. The duration of the signal above the difficulty level (circled part) corresponded to the rewarded effort duration.

Understanding how individuals with schizophrenia adapt their effort in real time in response to immediate demands and fatigue is crucial for elucidating the mechanisms underlying their difficulties with effort allocation in daily life. Here, we employed a task that allowed participants to freely allocate effort or rest while knowing that their reward would be proportional to the duration of their effort (Meyniel et al., 2013). Participants were required to squeeze a handgrip to accumulate as much money as possible during a 30-s epoch (Fig. 1A). Unlike previous effort tasks, the reward was determined by the duration spent maintaining a force level above a target force level (difficulty). This difficulty level was manipulated in a three-by-three design to vary the perceived cost of obtaining a reward: monetary incentive (0.2€, 1€, or 2€) and effort difficulty (60 %, 65 %, or 70 % of the subject's maximal force). We applied this behavioral paradigm to investigate the hypothesis that schizophrenia is associated with impairments in several dynamic cost/benefit decision-making processes, including (1) the adaptation of effort and rest durations across difficulty and incentive conditions, (2) the re-initiation of efforts across conditions, and (3) the dynamic adjustment of force level to maximize payoff.

## 2 Method

### 2.1 Participants

Participants included individuals with schizophrenia or schizoaffective disorder according to the DSM-5.0 (APA, 2013). Diagnoses were confirmed by a trained psychiatrist using the Structured Clinical Interview for DSM Disorders (SCID 2.0). Controls were free of current neurological or psychiatric disorder as assessed by the Mini International Neuro-psychiatric Interview (MINI) (Hergueta and Weiller, 2013). Inpatients were recruited from psychiatric hospitals in Grenoble, while outpatients were recruited through word-of-mouth and advertisements in healthcare centers. Healthy controls were recruited through word-of-mouth and on advertising platforms such as RISC and echoscience.

Pre-morbid IQ was assessed in both groups using the French version of the National Adult Reading Test (fNART) (Bright et al., 2018). Depressive symptoms, negative symptoms, subjective quality of life, insight and cognitive abilities were measured in the schizophrenia group using the following assessments validated in French language: Positive and Negative Syndrome Scale (PANSS) (Kay et al., 1987), Brief Negative Symptom Scale (BNSS) (Mucci et al., 2015), Self-evaluation of Negative Symptom (SNS) (Dollfus et al., 2016), Beck Depression Inventory (BDI) (Jackson-Koku, 2016), the Calgary Depression Scale for Schizophrenia (CDSS) (Addington et al., 1993), Subjective Quality of Life (SQoL) (Boyer et al., 2010), Beck Cognitive Insight (BCI) (Beck et al., 2004) and Subjective Scale to Investigate Cognition in schizophrenia (SSTICS) (Stip et al., 2003).

### 2.2 Dynamic effort allocation task

Subject force was recorded using a hand dynamometer (Vernier HD-BTA) at a sampling rate of 60 Hz. The task included three blocks of 18 trials, with varying conditions: three levels of difficulty (60 %, 65 %, 70 % of maximal force), three levels of incentive (0.2€, 1€, 2€), and alternating hands (left, right). Participants alternated hands between trials. The task was programmed using MATLAB 2018a and the PsychToolBox 3 psychophysics package (Kleiner et al., 2007).

Prior to the task, participants' maximal force was recorded using the Cléry-Melin et al. (2011) procedure. During three 5-s trials in each hand, they were instructed to squeeze the handgrip as hard as possible, with verbal encouragement to exert greater force in each trial. The highest force value obtained for each hand was then used to calibrate the effort levels during the task, ensuring that thresholds were individualized and hand-specific (i.e., accounting for potential differences between dominant and non-dominant hands). A training session of six trials followed the six calibration trials. The training trials were designed to maximize participants' understanding of the task while avoiding physical fatigue. We combined three low difficulty levels (15 %, 20 %, 25 % of the maximal force) and three incentive levels (0.2€, 1€, 2€) with short, low-intensity duration (10 s).

Participants then completed the three blocks of 18 experimental trials. Each trial began with a 2-s presentation of the incentive level coin and difficulty level. Participants had 30 s to gain as much money as possible by maintaining their force level above the displayed difficulty bar. Real-time feedback on the force exerted and the current amount of monetary gain accumulated were continuously displayed on the screen during each 30 s epoch of a trial. At the end of each trial, feedback on the cumulated amount of money won from the beginning of the block was given simultaneously with the gains obtained during the current trial. On each trial, a break option of up to 30 s was also presented. If a subject took the full break, the next trial would automatically start after a warning message (Fig. 1A).

Recorded data for analysis included the hand used, incentive level, difficulty level, money won, accumulated money, time spent above the difficulty target, intertrial break time, and the dynamometer force signal.

### 2.3 Data analyses

Statistical analyses were conducted using R.4.4.1 (RStudio, 2020). Mixed-effects ANOVA analyses were performed using the *lme4* (Bates et al., 2023) and the contrast analyses were performed using the *emmeans* package (Searle et al., 1980). Contrasts were examined using Tukey tests. Correlational analyses between variables were conducted using Pearson *r* parametric testing. The level of significance was 0.05.

We employed the offline epoching algorithm from Meyniel et al. (2013) to identify effort and rest periods within each trial (Fig. 1B). Tentative effort onsets were marked when the temporal derivative of the force signal was positive and the force level exceeded 30 % of the maximal force. Tentative offsets were identified with opposite criteria: negative temporal derivative and force level below 30 % of the maximal force. When multiple tentative offsets occurred between two tentative onsets, only the offset with the lowest force level was retained if the signal between this last offset and the previous onset contained time points above 30 % of the maximal force. Otherwise, the last tentative offset was kept. Additionally, if the trial ended during a sustained effort, the trial's end was marked as an offset. Effort and rest durations were calculated as the duration between onsets and offsets within each trial. The first resting period was considered to start at the coin presentation, as it was part of effort preparation.

Mixed-model ANOVAs were conducted to examine group (schizophrenia vs. healthy controls), incentive (0.2€, 1€, 2€), and difficulty (60 %, 65 %, 70 % of maximal force) effects on dependent variables.

Dependent variables included (1) single effort durations, (2) single rest durations, (3) number of effort re-initiations, defined as the number of distinct single effort within a trial (computed as the total number of single efforts minus one, to reflect the number of times the participant resumed an effort after a rest), and (4) rewarded effort duration (total duration of single effort spent above the difficulty bar during a trial). Each dependent variable was analyzed in separate mixed-model ANOVAs. Maximal force was included as a covariate to account for group differences, while trial index and block index were z-scored and added as covariates to control for subject's fatigue.

## 3 Results

Fifty-five participants were recruited, including 20 patients with schizophrenia, 8 patients with schizoaffective disorder and 27 healthy controls. Five participants were excluded from the analyses due to non-compliance with instructions (*n* = 2; both SZ), insufficient maximal force (*n* = 1; SZ), or epoching misclassification (n = 2; both controls). All included participants (*n* = 25 patients, n = 25 controls) exhibited alternating effort and rest periods. Controls and individuals with schizophrenia had similar break durations between trials (*F*₍₁,₁₄₀₉₎ = 0.34, *P* = 0.56; η^2^ = 0.02). There were no significant group differences in age, pre-morbid IQ, education, or sex ratio. Controls exhibited higher maximal force than patients. Therefore, maximal force was included as a covariate in all statistical models to rule out the possibility that reduced sensitivity to reward and effort cost in individuals with schizophrenia could be merely attributed to differences in maximal force capacity (Table 1).

**Table 1:** Socio-demographic and clinical data across groups.

		Schizophrenia (*N* = 25)	Controls (N = 25)	Statistics^⁎^
Age		37.5 (2.5)	37.7 (2.7)	W = 314; *P* > 0.9
Sex (female:male)		9:16	6:19	χ^2^(1) = 0.4; *P* = 0.5
Education		12.9 (0.4)	12.6 (0.2)	W = 277; P = 0.5
Premorbid IQ (fNART)		104.6 (2.0)	109.1 (1.7)	W = 402; *P* = 0.09
Maximal force in Newton (s.e.m)	Dominant hand	194.8 (17.1)	287.6 (13.4)	t(52) = 4.6; P < 0.001
	Non-dominant hand	172.3 (14.5)	261.9 (15.1)	t(52) = 4.5; P < 0.001
Negative symptoms (s.e.m)	PANSS negative	21.0 (1.4)	N.A	N.A.
	PANSS amotivation^#^	6.2 (0.7)		
	BNSS	27.0 (3.4)		
	BNSS amotivation^∼^	3.7 (0.7)		
	SNS	20.4 (1.5)		
Chlorpromazine equivalent (mg)		437.9 (53.6)	N.A	N.A.

Continuous data are presented as mean ± standard error of the mean. fNART: French national adult reading test, PANSS: Positive and Negative Syndrome Scale, BNSS: Brief Negative Symptom Scale, SNS: Self-reported Negative Symptoms scales. ^⁎^: *t*-test if continuous normally distributed data or Wilcoxon test if continuous non-normally distributed data or chi square if comparison of proportion. ^#^: items N2 and N4 (Strauss et al., 2021); ^∼^: items 7 and 8 (Strauss et al., 2021).

Each participant completed three blocks of the dynamic effort allocation task. The force signal of each subject was extracted and epoched into effort and rest periods using a methodology previously validated (Meyniel et al., 2013; see methods). In the following, we compared (1) the mean durations of single effort and rest periods, (2) the number of effort re-initiations and (3) the rewarded effort duration (i.e., the cumulative time epochs during which the grip signal was above the difficulty threshold allowing participants to accumulate money, indicating sustained and effective exertion (see Fig. 1B).

### 3.1 Dynamic effort allocation

#### 3.1.1 Effort and rest duration

**Fig. 2:**
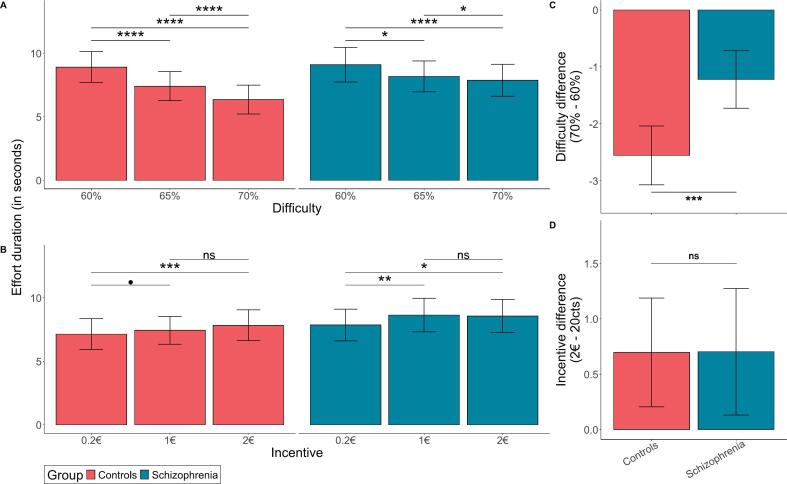
**Single effort duration across difficulty and incentive conditions. A:** Data are averaged per incentive levels. **B:** Data are averaged per difficulty levels. **C.** Plot of the interaction effect between group and difficulty on effort duration. Bars represent the effort duration difference between the highest difficulty (70 % of maximal force) and the lowest difficulty (60 % of the maximal force). **D.** Plot of the interaction effect between group and incentive on effort duration. Bars represent the effort duration difference between the highest incentive (2€) and the lowest incentive (20cts). The stars represent the *p*-value of the group difference between levels of difficulty and incentive. The intervals on the bars represent the standard error of the mean. ****: *p* < 0.0001; ***: *p* < 0.001; **: *p* < 0.01; *: *p* < 0.05; •: *p* < 0.1; ns: non-significant.

Controls decreased single effort durations with higher difficulty levels (*F*_(2,11__,__738.25)_ = 64.4, *p* < 0.001; η^2^ = 0.01; Fig. 2A) and increased single effort durations with higher incentives (*F*_(2,11__,__738.81)_ = 6.9; η^2^ = 0.001, *p* = 0.001; Fig. 2B). Contrary to our hypothesis, no main effect of group was observed on single effort duration (Effort duration: *F*_(1,49)_ = 0.06, *p* = 0.81; η^2^ = 0.001). However, we found a group-by-difficulty interaction (Effort duration: *F*_(2,11__,__739)_ = 9.9, *p* = 5.1 × 10^−5^; η^2^ = 0.002; Fig. 2C), indicating that individuals with schizophrenia exhibited a smaller decrease in effort duration between difficulty levels compared to controls (difference 70 %–60 %: *t*_*(49)*_ = 4.4, *p* = 0.0007; Cohen's d = 1.25; Fig. 2C). We found no group-by-incentive interaction (Effort duration: *F*₍₂,₁₁₇₃₉₎ = 0.96, *p* = 0.38; η^2^ = 0.0002; Fig. 2D).

**Fig. 3:**
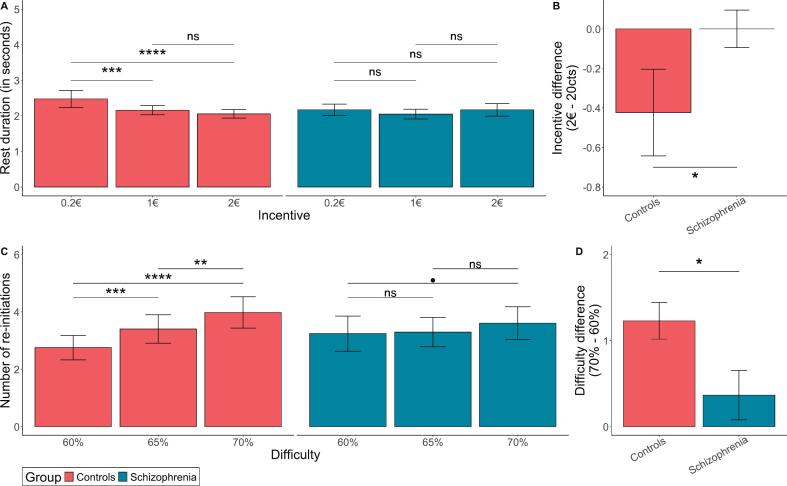
**Single rest duration and number of effort re-initiations across incentive and difficulty conditions. A.** Data averaged per difficulty level. **B.** Interaction effect between group and incentive on rest duration. Bars represent the difference in rest duration between the highest (2€) and lowest (0.2€) incentive levels. Stars represent the p-value of the group difference between levels of difficulty and incentive. Error bars indicate the standard error of the mean. ****: p < 0.0001; ***: p < 0.001; *: p < 0.05; ns: non-significant. **C:** Data averaged per incentive level. **D:** Interaction effect between group and difficulty on the number of effort re-initiations. Bars represent the difference in the number of re-initiations between the highest (70 %) and lowest (60 %) difficulty levels. Stars represent the p-value of the group difference between levels of difficulty and incentive. Error bars indicate the standard error of the mean. ****: p < 0.0001; ***: p < 0.001; **: p < 0.01; *: p < 0.05; •: p < 0.1; ns: non-significant.

Controls also decreased single rest durations with higher incentives (*F*_(2,11__,__906.32)_ = 15.3, p < 0.001; η^2^ = 0.0001; Fig. 3A). Contrary to our hypothesis, no main effect of group was observed on single rest duration (Rest duration: *F*_(1,54)_ = 0.12, *p* = 0.73; η^2^ = 0.002). Additionally, we observed a significant group-by-incentive interaction on rest duration (Fig. 3B). Rest duration was not significantly affected by incentive levels in the schizophrenia group compared to controls (difference 2€ – 20cts: *t*_(55)_ = 3.2, *p* = 0.02; Cohen's d = 0.91; Fig. 3B). These findings suggest that individuals with schizophrenia did not parametrically vary the duration of effort or rest durations in response to difficulty and incentive levels as effectively as controls.

#### 3.1.2 Self-initiated effort re-initiation

We next examined the number of effort re-initiations, as clinical accounts of schizophrenia suggest an existing deficit in real-life effort re-initiation. Yet, contrary to this clinical intuition, controls and individuals with schizophrenia exhibited similar numbers of re-initiated efforts across trials (*F*_(1,53)_ = 0.25, *p* = 0.62;; η^2^ = 0.005). However, a significant group-by-difficulty interaction was observed (*F*_(2,2629)_ = 6.6, *p* = 0.001; η^2^ = 0.005; Fig. 3C), indicating that controls increased effort re-initiations with higher difficulty levels (all p's < 0.01) to a greater extent than individuals with schizophrenia (all p's > 0.05; difference 70 %–60 %: *t*_(54)_ = 3.5, *p* = 0.01; Cohen's d = 0.99; Fig. 3D). These findings suggest that schizophrenia is associated with suboptimal effort re-initiation patterns in response to increasing difficulty levels, highlighting a specific deficit in dynamic adaptive allocation of effort strategies that has not been previously studied.

#### 3.1.3 Rewarded effort duration

**Fig. 4:**
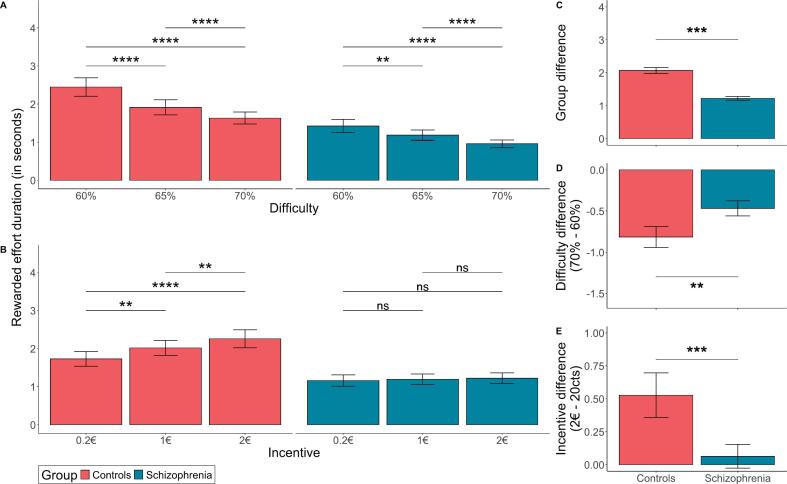
**Rewarded effort duration across difficulty and incentive conditions A:** Data aggregated across difficulty and incentive levels. **B.** Interaction effect between group and difficulty on rewarded effort duration. Bars represent the difference in rewarded effort duration between the highest (70 %) and lowest (60 %) difficulty levels. **C.** Interaction effect between group and incentive on rewarded effort duration. Bars represent the difference in rewarded effort duration between the highest (2€) and lowest (0.2€) incentive levels. Stars represent the p-value of the group difference between levels of difficulty and incentive. Error bars indicate the standard error of the mean. ****: p < 0.0001; ***: p < 0.001; *: p < 0.05; ns: non-significant.

We examined the duration participants spent exerting force above the target difficulty level (i.e., the duration during which they accumulated money). Individuals with schizophrenia spent less time accumulating money than controls across all difficulty and incentive levels (F₍₁,₅₃₎ = 14, *P* = 0.00041; η^2^ = 0.17; Fig. 4C). Both groups increased rewarded effort duration with higher incentives (F₍₂,₂₀₇₀₇₎ = 18.1, *P* = 1.5 × 10^−8^; η^2^ = 0.003; Fig. 4B) and decreased it with higher difficulty levels (F₍₂,₂₀₇₀₆₎ = 89.8, P = 1.5 × 10^−39^; η^2^ = 0.005; Fig. 4A). In summary, individuals with schizophrenia spend less time accumulating money but have similar effort duration, which is explained by a lower proportion of their efforts reaching the difficulty level, indicating suboptimal effort allocation.

#### 3.1.4 Relationships of effort allocation to clinical variables

No significant correlations were found between output performance metrics, antipsychotic equivalent dosage, and negative symptoms, including motivational negative symptoms (all *p*-values >0.05). No significant group differences were found between high and low amotivation using the PANSS and the BNSS (all p-values >0.05). We also examined correlations between the behavioral variables and subcomponents of the BNSS and the SNS, in accordance with their established 5-factors structure. No significant associations were found. These results are detailed in Supplementary Table S1.

## 4 Discussion

In this study, we employed a dynamic effort allocation task in a sample of individuals with schizophrenia that were compared to healthy controls. Participants were required to dynamically adapt their effort duration, effort re-initiation, and rest duration to optimize effort allocation and maximize gains. Our main findings were as follows: (1) Maladaptive effort allocation: patients showed difficulties in adjusting their effort and rest durations to varying task demands, such as changes in difficulty level or incentive levels. (2) Intact effort re-initiation: despite these challenges, patients did not differ from controls in terms of their overall effort duration or their ability to re-initiate effort. (3) Reduced monetary gain: patients with schizophrenia gained less money due to shorter rewarded effort durations; particularly there was a reduced adaptation to difficulty and incentive levels.

Individuals with schizophrenia utilized difficulty information less optimally to guide in-the-moment effort allocation compared to controls. While rest durations were not significantly different between groups, individuals with schizophrenia failed to adapt rest durations to incentive levels. This adds to the growing body of evidence suggesting a disrupted effort allocation process in schizophrenia (Blouzard et al., 2023; Gold et al., 2015; Saleh et al., 2023). Moreover, the results suggest that abnormal effort allocation over time may arise from a poor recovery process, such as the failure to adjust rest durations based on incentive levels. This deficit may contribute to the daily life struggles of patients, particularly in terms of re-initiation adaptability. Future research is needed to investigate the origins of disrupted effort-cost computations in schizophrenia. These may originate at early stages of incentive and difficulty presentation, with downstream consequences on online adaptation. Alternatively, they may emerge during online adaptation, such as through the overestimation of fatigue. Further exploration of these possibilities is also warranted.

The present study is the first to disentangle the dynamic interplay of perceived costs and benefits on effort in schizophrenia. Lower rewarded effort durations in the schizophrenia group suggest a deficit in effort allocation. While rewards energize behavior in schizophrenia (Strauss and Gold, 2012), the deficit in difficulty adaptation is likely not due to a lack of task comprehension, as both groups exhibited similar patterns (though not similar in magnitude) of adaptation in rewarded effort duration. This aligns with the established finding that individuals with schizophrenia do not present with a deficit in consummatory pleasure but rather in the anticipation of pleasure (motivational anhedonia), consistent with observations that higher rewards fail to invigorate effort during rest periods in patients (Strauss and Gold, 2012). Unlike static effort-cost decision-making paradigms, the deficit in difficulty adaptation was not specific to rest and reward. Individuals with schizophrenia also increased their rewarded effort duration and single effort duration for higher rewards less than controls. This suggests that reward devaluation does not contribute to faulty effort allocation in patients.

Another key finding of our study is that individuals with schizophrenia exhibited significantly impaired ability to adjust their effort based on task difficulty. Deficits in this process may contribute to abnormal effort allocation in schizophrenia. This result corroborates Meyniel's model, in which task difficulty influences real-time effort adaptation, while monetary incentives can be considered strategic adjustments during rest periods to facilitate re-initiation of effort (Meyniel et al., 2013). These findings align with prior computational studies identifying a subgroup of patients with impaired integration of probability and reward information in effort-based decision-making tasks (Cooper et al., 2019; Gold et al., 2015). Our results extend this framework by suggesting an additional deficit in assessing task difficulty and optimizing effort-rest allocation over time. This may reflect a broader inefficiency in processing and integrating task-relevant information. Future studies with larger samples will be essential to apply computational modeling approaches to this paradigm, in order to isolate the specific mechanisms underlying the cost–effort abnormality reflected in suboptimal effort–rest allocation over time. Our results fit within the framework proposed by Gold and colleagues (Gold et al., 2008), which emphasizes a core deficit in schizophrenia related to representing reward value. Our data suggest that while individuals with schizophrenia may have impaired integration of overall incentive signals as reflected by the lack of modulation of rest duration over time, they retain some capacity for immediate, “in-the-moment”, reward evaluation, as shown by increased effort duration when incentives are presented. This dissociation highlights a nuanced impairment in cost-effort computations, where the anticipation or representation of reward value is impaired, but moment-to-moment motivational drive can still influence behavior. Understanding this distinction could inform targeted interventions aiming to enhance reward representation and improve motivational deficits in schizophrenia.

Our findings suggest that individuals with schizophrenia exhibit a reduced sensitivity to in-the-moment difficulty levels when deciding whether to cease or resume effort. Additionally, the speed of recovery from effort exertion during rest may not be guided by incentive levels in patients, indicating a failure to re-energize behavior when needed. This idea is supported by data showing an accumulation/dissipation signal that originates from interoceptive thalamo-insular structures, which are known to be altered in schizophrenia (Bora et al., 2011). Hence, we propose that dysregulated signals from proprioceptive regions associated with aberrant neurotransmission might contribute to effort allocation deficits in schizophrenia. Among neurotransmitters implicated in effort-based decision-making, serotonin has been shown to reduce the subjective cost of effort and promote higher payoffs, while dopamine has been consistently associated with enhancing the incentive salience of potential rewards in the healthy brain (Meyniel et al., 2016; Pessiglione et al., 2006; Wardle et al., 2011). However, the interpretation of dopaminergic contributions remains complex. For example, studies using a mouse model of striatal D2 receptor overexpression have demonstrated that motivational deficits—rather than primary cognitive impairments—can disrupt the ability to use reward-predictive cues to guide attention. These findings underscore the importance of motivational impairments as a limiting factor for cognitive performance, suggesting that dynamic interactions between motivation and cognition may underlie functional outcomes in schizophrenia (Ward et al., 2015). Noradrenaline has also been implicated in energizing behavior and engaging effort (Varazzani et al., 2015). Decades of research have suggested roles for these neurotransmitters in the pathophysiology of motivational impairments in schizophrenia (Brasso et al., 2023; Mäki-Marttunen et al., 2020; Risch, 1996), although their precise involvement in impaired effort allocation remains to be investigated. Another possibility is that aberrant glutamatergic neurotransmission in schizophrenia (Dondé et al., 2023; Javitt et al., 2012) could contribute to abnormal effort allocation. Wiehler et al. (2022) found that glutamate indexes daylong fatigue and is implicated in representing increases in effort cost as fatigue increases. Glutamatergic signaling or higher basal levels of glutamate in schizophrenia might contribute to higher perceived effort costs, disrupting fatigue accumulation/perception and engagement in daily-life actions.

We acknowledge several limitations. First, the schizophrenia group exhibited lower maximal force, which may have influenced the lack of adaptation to effort difficulty. However, our statistical models controlled for maximal force, indicating that the task did not require less effort for this group. The lower maximal force could be attributed to psychomotor slowing, a core feature of schizophrenia resulting from cognitive planning impairments (Osborne et al., 2020). This might also explain the lack of difficulty adaptation, as perseverance during unrewarded effort periods could lead to higher fatigue levels in individuals with schizophrenia and further impair effort allocation. Psychomotor slowing and negative symptoms are overlapping manifestations of schizophrenia and may together contribute to suboptimal effort adaptation (Peralta et al., 2018). Reduced physical strength, consistently reported in schizophrenia and reliably indexed by grip strength, may also contribute to altered patterns of dynamic effort allocation (Dlagnekova et al., 2021).

Second, effort-cost decision-making has been repeatedly found to be mediated by general cognitive performance, which was not measured in our study (Cooper et al., 2019; Foussias et al., 2014). However, our task did not require explicit choices, limiting the potential cognitive contribution to effort allocation. Finally, we did not find any significant relationship between effort measures and motivational negative symptoms, contradicting previous results of impaired effort allocation in higher versus lower motivational symptoms samples (Blouzard et al., 2023). Given the small sample size, we cannot rule out the possibility that a significant association between motivational symptoms and effort allocation exists but was not detected in this study. A sample size of 25 is likely underpowered, as detecting a moderate bivariate correlation (r ≈ 0.5) with 80 % power at α = 0.05 typically requires approximately 30 participants. Future research in larger samples will be essential to determine whether reliable associations emerge under specific conditions.

Effort allocation impairments are well-documented behavioral deficits in schizophrenia. This study represents the first investigation of dynamic effort allocation in patients, employing a paradigm with greater ecological validity for studying effort allocation in schizophrenia. Individuals with schizophrenia demonstrated suboptimal effort allocation, taking limited account of task difficulty during effort production and re-initiation. This deficit may contribute to maladaptive behavior patterns characterized by suboptimal effort allocation and reduced goal-direct activities.

The following is the supplementary data related to this article.

Table S1 Correlation between amotivation and effort allocation variables. Table S1

## CRediT authorship contribution statement

**Elodie Blouzard:** Writing – review & editing, Writing – original draft, Visualization, Software, Project administration, Methodology, Investigation, Formal analysis, Data curation. **Fabien Cignetti:** Writing – review & editing, Validation. **Florent Meyniel:** Writing – review & editing, Resources, Methodology, Conceptualization. **Arnaud Pouchon:** Writing – original draft. **Mircea Polosan:** Writing – review & editing, Methodology, Conceptualization. **Julien Bastin:** Writing – original draft, Validation, Supervision, Software, Project administration, Methodology, Investigation, Funding acquisition, Conceptualization. **Clément Dondé:** Writing – review & editing, Validation, Supervision, Resources, Project administration, Methodology, Investigation, Funding acquisition, Conceptualization.

## Role of the funding source

The funding source had no role in the study design, collection, analysis and interpretation of data, writing of the report and decision to submit the article for publication.

## Declaration of competing interest

None reported.
