# Supplementary material for: Impaired effort allocation in schizophrenia
Source: Schizophr Res Cogn. 2025 Jul 15;42:100378. doi: 10.1016/j.scog.2025.100378 (PMC12282204; doi:10.1016/j.scog.2025.100378)
Supplement: Table S1 — Correlation between amotivation and effort allocation variables. [file mmc1.docx]

# Impaired effort allocation in schizophrenia

Elodie **BLOUZARD**^1^, Fabien **CIGNETTI**^1^**,** Florent **MEYNIEL**^2, 3^**,** Arnaud **POUCHON**^1,4^**,**

Mircea **POLOSAN**^1,4^, Julien **BASTIN***^1^**,** Clément **DONDÉ***^1,4,5^

*****contributed equally to this manuscript

^1^ Univ. Grenoble Alpes, Inserm, U1216, CHU Grenoble Alpes, Grenoble Institut Neurosciences, 38000 Grenoble, France

^2^ INSERM-CEA Cognitive Neuroimaging Unit (UNICOG), NeuroSpin Center, CEA Paris-Saclay, Gif-sur-Yvette, France Université de Paris, Paris, 91191 France

^3^ Institut de Neuromodulation, GHU Paris, Psychiatrie et Neurosciences, Centre Hospitalier Sainte-Anne, Pôle Hospitalo-Universitaire 15, Université Paris Cité, Paris, 75015 France

^4^ Adult Psychiatry Department, Centre Hospitalier Alpes-Isère, F-38000 Saint-Egrève, France.

^5^ Adult Psychiatry Department, CHU Grenoble Alpes 38000 Grenoble, France

**Running title:** Impaired effort allocation in schizophrenia

***Corresponding Author**

Clément DONDÉ, MD PhD

Department of Psychiatry, CH Alpes-Isère, Pôle hospitalo-universitaire, bât. 21, 3 rue de la Gare, 38120 Saint-Egrève, FRANCE

e.mail : clement.donde@univ-grenoble-alpes.fr /

Tel: 04. 76. 76. 39. 86 / 88. 57

https://orcid.org/0000-0002-5121-8769

**Supplementary tables**

**Table S1: Correlation between amotivation and effort allocation variables.**

|  | Effort duration | Rest duration | Effort  re-initiations | Rewarded effort duration |
| --- | --- | --- | --- | --- |
| PANSS Amotivation | r = -0.11;  P = 0.61 | r = -0.22;  P = 0.29 | r = -0.02;  P = 0.91 | r = 0.16;  P = 0.46 |
| BNSS Total | r = -0.01;  P = 0.96;  P adj = 0.96 | r = 0.07;  P = 0.78;  P adj = 0.95 | r = -0.07;  P = 0.80;  P adj = 0.96 | r = -0.12;  P = 0.64;  P adj = 0.88 |
| BNSS Amotivation | r = 0.08;  P = 0.74;  P adj = 0.96 | r = 0.06;  P = 0.82;  P adj = 0.95 | r = -0.27;  P = 0.29;  P adj = 0.96 | r = 0.07;  P = 0.81;  P adj = 0.88 |
| BNSS Anhedonia | r = -0.11;  P = 0.68;  P adj = 0.96 | r = -0.06;  P = 0.83;  P adj = 0.95 | r = 0.05;  P = 0.84;  P adj = 0.96 | r = -0.38;  P = 0.14;  P adj = 0.55 |
| BNSS Expressivity | r = 0.10;  P = 0.69;  P adj = 0.96 | r = 0.01;  P = 0.95;  P adj = 0.95 | r = 0.01;  P = 0.96;  P adj = 0.96 | r = 0.04;  P = 0.88;  P adj = 0.88 |
| BNSS Asociality | r = 0.13;  P = 0.59;  P adj = 0.93 | r = 0.01;  P = 0.92;  P adj = 0.95 | r = 0.01;  P = 0.88;  P adj = 0.96 | r = 0.04;  P = 0.89;  P adj = 0.89 |
| BNSS Alogia | r = -0.12;  P = 0.69;  P adj = 0.96 | r = -0.04;  P = 0.83;  P adj = 0.94 | r = 0.05;  P = 0.88;  P adj = 0.89 | r = -0.32;  P = 0.15;  P adj = 0.53 |
| SNS Total | r = 0.36;  P = 0.16;  P adj = 0.46 | r = 0.15;  P = 0.56;  P adj = 0.73 | r = -0.51;  P = 0.03;  P adj = 0.10 | r = 0.15;  P = 0.56;  P adj = 0.56 |
| SNS Social withdrawal | r = 0.51;  P = 0.03;  P adj = 0.23 | r = 0.22;  P = 0.39;  P adj = 0.73 | r = -0.59;  P = 0.01;  P adj = 0.08 | r = 0.25;  P = 0.33;  P adj = 0.52 |
| SNS Diminished emotional range | r = 0.20;  P = 0.45;  P adj = 0.54 | r = -0.13;  P = 0.61;  P adj = 0.73 | r = -0.20;  P = 0.45;  P adj = 0.45 | r = -0.24;  P = 0.35;  P adj = 0.52 |
| SNS Alogia | r = 0.14;  P = 0.59;  P adj = 0.59 | r = 0.06;  P = 0.80;  P adj = 0.80 | r = -0.29;  P = 0.26;  P adj = 0.31 | r = 0.17;  P = 0.52;  P adj = 0.56 |
| SNS Avolition | r = 0.20;  P = 0.44;  P adj = 0.54 | r = 0.27;  P = 0.29;  P adj = 0.73 | r = -0.44;  P = 0.08;  P adj = 0.12 | r = 0.41;  P = 0.11;  P adj = 0.52 |
| SNS Anhedonia | r = 0.31;  P = 0.23;  P adj = 0.46 | r = 0.14;  P = 0.59;  P adj = 0.73 | r = -0.48;  P = 0.05;  P adj = 0.10 | r = 0.30;  P = 0.24;  P adj = 0.52 |

PANSS: Positive and negative symptom scale (Kay, Fiszbein, & Opler, 1987). PANSS amotivation: N2 + N4 (Strauss, Bartolomeo, & Harvey, 2021); BNSS: Brief negative symptom scale (Mucci et al., 2015). BNSS amotivation: item 7 + item 8 (Strauss et al., 2021). SNS: Self-reported negative symptom (Dollfus, Mach, & Morello, 2016).

r: Pearson correlation coefficient ; P: p-value; P adj : FDR corrected p-value.

Bibliography

Dollfus, S., Mach, C., & Morello, R. (2016). Self-Evaluation of Negative Symptoms  : A Novel Tool to Assess Negative Symptoms. *Schizophrenia Bulletin*, *42*(3), 571–578. https://doi.org/10.1093/SCHBUL/SBV161

Kay, S. R., Fiszbein, A., & Opler, L. A. (1987). The positive and negative syndrome scale (PANSS) for schizophrenia. *Schizophrenia Bulletin*, *13*(2), 261–276. https://doi.org/10.1093/schbul/13.2.261

Mucci, A., Galderisi, S., Merlotti, E., Rossi, A., Rocca, P., Bucci, P., … Maj, M. (2015). The Brief Negative Symptom Scale (BNSS): Independent validation in a large sample of Italian patients with schizophrenia. *European Psychiatry*, *30*(5), 641–647. https://doi.org/10.1016/j.eurpsy.2015.01.014

Strauss, G. P., Bartolomeo, L. A., & Harvey, P. D. (2021). Avolition as the core negative symptom in schizophrenia: relevance to pharmacological treatment development. *Npj Schizophrenia*, Vol. 7. https://doi.org/10.1038/s41537-021-00145-4
